# Intensive formation of coccoid forms as a feature strongly associated with highly pathogenic *Helicobacter pylori* strains

**DOI:** 10.1007/s12223-018-0665-5

**Published:** 2018-11-17

**Authors:** Paweł Krzyżek, Monika M. Biernat, Grażyna Gościniak

**Affiliations:** 1 Department of Microbiology, Faculty of Medicine, Wroclaw Medical University, Wroclaw, Poland; 2 Department of Haematology, Blood Neoplasms, and Bone Marrow Transplantation, Faculty of Postgraduate Medical Training, Wroclaw Medical University, Wroclaw, Poland

## Abstract

The variability of *Helicobacter pylori* morphology and the heterogeneity of virulence factors expressed by these bacteria play a key role as a driving force for adaptation to the hostile stomach environment. The aim of the study was to determine the relationship between the presence of certain genes encoding virulence factors and *H. pylori* morphology. One reference and 13 clinical *H. pylori* strains with a known virulence profile (*vacA*, *cagA*, *babA2*, *dupA*, and *iceA*) were used in this study. Bacteria were cultured for 1 h and 24 h in stressogenic culture conditions, i.e., serum-free BHI broths at suboptimal conditions (room temperature and atmosphere, without shaking). *H. pylori* cell morphology was observed by light and scanning electron microscopy. The *vacA* polymorphism and the *cagA* and *babA2* presence were positively correlated with the reduction in cell size. Exposure to short-time stressogenic conditions caused more intense transformation to coccoid forms in highly pathogenic *H. pylori* type I strains (35.83% and 47.5% for type I s1m2 and I s1m1, respectively) than in intermediate-pathogenic type III (8.17%) and low pathogenic type II (9.92%) strains. The inverse relationship was observed for the number of rods, which were more common in type III (46.83%) and II (48.42%) strains than in type I s1m2 (19.25%) or I s1m1 (6.58%) strains. Our results suggest that there is a close relationship between the presence of virulence genes of *H. pylori* strains and their adaptive morphological features.

## Introduction

*Helicobacter pylori* is a microaerophilic, spiral, Gram-negative rod capable of effectively colonizing the gastric mucosa (Marshall and Warren 1984). Bacteria spread directly from person to person and are responsible for the development of a wide range of gastrointestinal diseases, including chronic (active) gastritis, gastric and/or duodenal ulcers, gastric adenocarcinomas, and mucosa-associated lymphoid tissue lymphomas (Malfertheiner et al. 2017). The presence of gastrointestinal pathologies and their severity is influenced by the type of bacterial strains and individual predispositions of the host, which determine the switching of *H. pylori* between commensalism and pathogenicity (Larussa et al. 2015). The long-lasting equilibrium between the host and *H. pylori*, covering at least 60,000 years, has enabled these microbes to develop evolutionary adaptations (Atherton and Blaser 2009; Talebi Bezmin Abadi 2017). The ability to avoid immune clearance and rapid response to changing environmental conditions are key determinants of the persistent host colonization by *H. pylori* (Larussa et al. 2015; Talebi Bezmin Abadi 2017).

One way of adaptation to environmental changes is the capability of *H. pylori* to undergo a morphological transformation. This microorganism occurs in two morphological forms, i.e., spiral—live, culturable, and coccoid—viable but nonculturable. The morphological transition from spiral to spherical form is observed under suboptimal environmental conditions, such as aerobiosis, temperature or pH changes, prolonged culture, or exposure to antibiotics or proton pump inhibitors (Loke et al. 2016). In proteomic studies, Loke et al. (2016) found that coccoid forms have high levels of proteins participating in DNA replication and carcinogenesis initiation and should therefore be regarded as highly virulent. The ability of *H. pylori* to produce elongated/ filamentous forms was also demonstrated, both in vitro (in *amiA* mutants and also after exposure to aztreonam, elevated NaCl concentrations or during oxygenated CO_2_-depleted culture) and in vivo (Takeuchi et al. 2006; Chaput et al. 2006; Park et al. 2011; Shao et al. 2012; Singh et al. 2015). It is postulated that increased cell filamentation may be a property of commensal bacteria, while the reduction of elongation is a hallmark of pathogenicity (Rossetti et al. 2013).

The heterogeneity of *H. pylori* strains and the variability of virulence factors expressed by these bacteria play a key role in adaptation to the stomach environment of the host (Odenbreit et al. 2009). The production of CagA (cytotoxin-associated gene A) is a feature of more virulent *H. pylori* strains, because this oncoprotein is capable of modulating host cell physiology (initiation of increased motility and elongated shape, the so-called hummingbird phenotype), induction of carcinogenesis, and IL-8-dependent initiation of inflammation. The vacuolating cytotoxin A (VacA) is a 140-kDa polypeptide responsible for the host tissue damage associated with the formation of anion-selective pores in cell membranes, mitochondria disruption, and eukaryotic cell death (Roesler et al. 2014; Amieva and Peek 2016). The VacA toxicity is determined mainly by two variable regions, called signal (s1 and s2) and middle (m1 and m2). The *vacA*s1 strains have a higher cytotoxic activity and are responsible for a greater risk of developing gastrointestinal diseases (especially *vacA*s1m1) than non-vacuolating *vacA*s2 strains (Roesler et al. 2014). Based on the expression of these two virulence factors, *H. pylori* strains were classified into three groups, i.e., with the highest virulence potential, *cagA*^+^/*vacA*s1 (type I) and the lowest virulence, *cagA*^−^/*vacA*s2 (type II), and strains with intermediate virulence with genotype, *cagA*^−^/*vacA*s1 or *cagA*^+^/*vacA*s2 (type III) (Xiang et al. 1995). Blood group antigen-binding adhesin A (BabA) is a protein associated with adhesion to the gastric mucosa, by binding to various components of the host (Ansari and Yamaoka 2017). Duodenal ulcer promoting gene A (DupA) is one of many important virulence factors often referred to as important in the promotion of gastrointestinal diseases. The production of this protein is coupled positively with the secretion of proinflammatory cytokine IL-8 (Queiroz et al. 2011). Another important virulence determinant involved in the pathogenicity of *H. pylori* is induced by contact with epithelium gene A (IceA). Production of this protein is stimulated by contact with the gastric epithelium, which contributes to the increase in IL-8 secretion (Shiota et al. 2012).

The aim of the study was to determine the relationship between the presence of certain genes encoding virulence factors and morphological changes of *H. pylori* strains.

## Materials and methods

### Bacterial strains and growth conditions

Virulence genes of all 13 clinical *H. pylori* strains used in this study (Table 2) have been identified and published previously by Biernat et al. (2010, 2014). In these reports, we have shown that *H. pylori* with specific virulence factors (*cagA*, *vacA*, *babA2*, *dupA*, and *iceA* in Table 2) were associated with the induction of gastric mucosa inflammation and tissue destruction. Detection of *vacA*, *iceA1*, and *babA2* was carried out using multiplex PCR, whereas the *cagA* and *iceA2* genes were detected by single PCR. *H. pylori* strains used in the study were formerly isolated from antral biopsy specimens of patients with gastritis. In addition, the reference strain J99 (ATCC 700824) was used as a control strain. The genome sequence of *H. pylori* J99 is available at GenBank with the accession number AE001439.

Culture methods of *H. pylori* strains were performed according to Macegoniuk et al. (2017) with minor modifications. The frozen bacterial suspensions of clinical and reference *H. pylori* strains were stored at − 70 °C in tryptic soy broth (Oxoid) with 15% glycerol. After reviving from frozen stocks, bacterial strains were pre-cultured onto Columbia agar (Difco) enriched with 7% hemolysed horse blood and *H. pylori*-selective supplement (Oxoid). Examined strains were incubated under microaerophilic conditions (Genbox microaer kits, BioMerieux) at 37 °C for 3 days and then sub-cultured on the same fresh media and incubated for 3 more days under the same conditions. In all of the following experiments, each *H. pylori* strain was passaged for only two or three times to reduce the risk of phase-variable changing of virulence genes (Odenbreit et al. 2009).

### Preparation of cultures for light microscopy

*H. pylori* strain J99 was used to find the best environment that yielded the lowest average cell length and highest percentage of coccoid forms. Bacteria from solid cultures were inoculated into 2 ml of fresh brain heart infusion (BHI, Sigma) broths to obtain OD_600_ of 0.3 (early log phase) or 1 (stationary phase). Each bacterial culture was then incubated for 1 h and 24 h under specified conditions: BHI with 7% fetal bovine serum (Gibco) at optimal conditions (37 °C, microaerophilic, 100 rpm), BHI without the serum at optimal conditions, BHI with the serum at suboptimal conditions (room temperature and atmosphere, without shaking), and BHI without the serum at suboptimal conditions.

### Cell length, shape determination, and image acquisition

Light microscopy analysis was performed similarly as described elsewhere (Rossetti et al. 2013) with small modifications. For each time point and strain, 50 μL of bacterial suspension was dropped onto a coverslip, stained by Gram’s method and observed under an Olympus BX50 microscope (Olympus Optical) using a × 100 oil immersion objective with numerical aperture of 1.3. The measurement of cell length was performed manually using the CellTool software. The average length of bacterial cells was counted using two independent experiments. From each experiment, three slides were taken and 50 bacterial cells per slide were counted (*n* = 300 cells/strain for each time point).

**Fig. 1:**
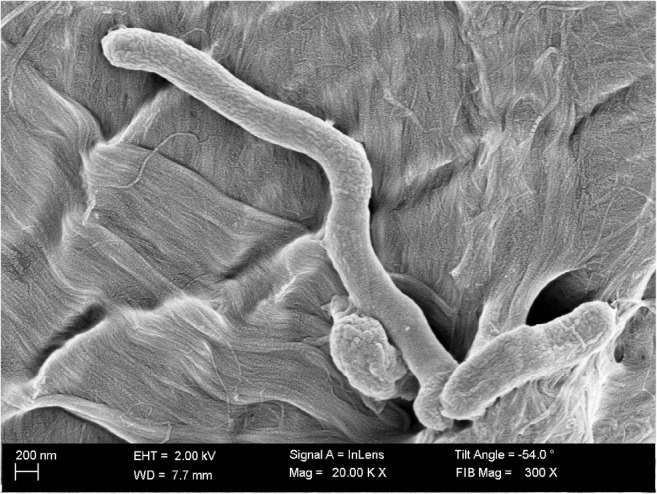
Morphological forms of *H. pylori* observed by scanning electron microscopy. The picture shows, from left to right, coccoid form, filamentous form, and rod

*H. pylori* cell forms were classified into five groups: coccoid forms (0.5–1 μm), short rods (1–2 μm), rods (2–4 μm), elongated rods (4–5 μm), and filamentous forms (≥ 5 μm) (Fig. 1). Classification of *H. pylori* cell length was determined by the observations of others (Nayak and Rose 2007; Hirsch et al. 2012; Chiou et al. 2013). Spherical forms larger than 1 μm, and rods shorter than 1 μm, accounted for less than 1% of the total pool of bacteria and did not affect the final results.

### Scanning electron microscopy

In order to precisely determine the morphological structure of *H. pylori* cells and to confirm observations made using light microscopy, scanning electron microscopy (SEM) was used. The morphology of two *H. pylori* strains with the greatest variation in the virulence profile, namely 6171/T.II (*vacA*s2m2, *cagA*^−^, *dupA*^−^, *iceA*^−^, *babA2*^−^) and J99 (*vacA*s1m1, *cagA*^+^, *dupA*^+^, *iceA2*^+^, *babA2*^+^), was analyzed by SEM. These strains from solid cultures were inoculated into 2 ml of fresh BHI (Sigma) broths to obtain OD_600_ of 0.3 (early log phase) and were cultured for 1 h in serum-free BHI broths at suboptimal conditions (room temperature and atmosphere, without shaking). After this time point, microbes in liquid media were prefixed in 2.5% glutaraldehyde for 24 h and pelleted by centrifugation (600*g*, 5 min) in microcentrifuge tubes. Pellets were washed a total of three times in 0.1 M cacodylate buffer (pH 7.4) after each step cetrifugating bacteria again (600*g*, 5 min). Microbes were treated with a series of ethanol concentrations (50, 70, 90, 99.99%) and pelleted by centrifugation (600*g*, 5 min). After dehydration in a graded series of ethanol, the specimens were coated with 10 nm of gold. The samples were examined with a scanning electron microscope (Auriga 60, Zeiss) operating at 2 kV.

### Statistical analysis

From two independent experiments, six slides per strain were taken and results were expressed as the mean ± standard deviation of the mean (SDM). All data were analyzed by STATISTICA v. 10.0. The distribution normality of continuous variables was calculated using the Shapiro-Wilk test. As data from bacterial cell sizes were not normally distributed, the correlation between variables was analyzed by the nonparametric Spearman’s rho. The differences in statistical significance between bacterial cell sizes at 1-h and 24-h incubation periods were analyzed by the Wilcoxon test. Cell morphology type frequencies were estimated by the Pearson *χ*^2^ analysis. The reduction of bacterial cell size after 1-day incubation period for *H. pylori* strains with specific virulence factors was analyzed by the Mann-Whitney *U* test and the Kruskal-Wallis test. The differences were found to be statistically significant at a *p* ≤ 0.05.

## Results

### Optimization of conditions inducing *H. pylori* morphological changes

After 1-h and 24-h incubation periods, there were no significant differences in the cell length distribution of *H. pylori* J99 in broths with different initial densities of bacteria, but at a density of OD_600_ = 1 bacterial cells were intensively autoaggregated (data not shown). Therefore, further studies were carried out using a density of OD_600_ = 0.3 to accurately determine the size and shape of a single bacterial cell.

It was observed that the reduction of *H. pylori* J99 in size and the changes in morphology were most prominent in serum-free BHI media at suboptimal culture conditions (Table 1); these counted for 1.02 ± 0.02 μm (56.3% coccoid forms and 42.3% short rods) and 0.9 ± 0.04 μm (71% coccoid forms and 29% short rods) at 1-h and 24-h incubation, respectively. Average bacterial cell length was highest in BHI broths with the serum at optimal culture conditions and equaled 2.26 ± 0.02 μm (70.3% rods and 26.7% short rods) after 1 h and 2.16 ± 0.04 μm (60% rods and 34.7% short rods) after 1 day of incubation periods. Other culture conditions contributed to the formation of intermediate-sized cells, i.e., 1.81 ± 0.04 and 1.45 ± 0.03 μm (suboptimal culture conditions, BHI with the serum) and 1.47 ± 0.03 and 1.06 ± 0.02 μm (optimal culture conditions, serum-free BHI) at 1-h and 24-h incubation, respectively.

**Table 1:** The impact of culture conditions on average cell length of *H. pylori* reference strain J99

Stressogenic factors		Average cell length ± SDM (min-max length) (μm)		
Serum starvation	Suboptimal culture conditions	0-h incubation	1-h incubation	24-h incubation
		2.1 ± 0.03 (0.7–3.98)	2.26 ± 0.02 (0.67–4.31)	2.16 ± 0.04 (0.68–8.28)
	+		1.81 ± 0.04 (0.54–3.88)	1.45 ± 0.03 (0.52–3.16)
+			1.47 ± 0.03 (0.52–4.85)	1.06 ± 0.02 (0.5–3.14)
**+**	**+**		1.02 ± 0.02 (0.52–2.1)	0.9 ± 0.04 (0.5–1.85)

In column “stressogenic factors”: + indicates the presence of tested factor during culture of microbes; free space indicates the absence of tested factor during culture of microbes, and the presence of 7% fetal bovine serum in BHI media and optimal culture conditions (37 °C, microaerophilic, 100 rpm), respectively

For this reason, bacterial inoculations (OD_600_ = 0.3) were incubated in serum-free BHI broths at suboptimal culture conditions for 1 h and 24 h in the studies of the remaining *H. pylori* strains.

### Analysis of cell length variability

Light microscopy observation of tested *H. pylori* strains at 0 h showed no significant differences in average cell length (*p* > 0.05), which ranged from 2.49 ± 0.03 to 2.1 ± 0.03 μm (Table 2). After 1-h and 24-h incubation, the average cell length of tested *H. pylori* strains changed profoundly and ranged between 2.44 ± 0.05 and 1.02 ± 0.02 μm and between 1.5 ± 0.05 and 0.9 ± 0.04 μm, respectively. Despite the gradual gradation in average bacterial cell length, coupled with the increasing number of virulence genes, these differences were not statistically significant (*p* > 0.05) (data not shown).

**Table 2:** The impact of the virulence factors profile on the cell length distribution of *H. pylori* strains after 0-h, 1-h, and 24-h incubation periods in stressogenic culture conditions

*H. pylori* strains	*vacA* polymorphism	Profile of virulence factors				Average cell length ± SDM (min-max length) (μm)			Reduction of cell length^†^ (%)		The Wilcoxon *Z* test; *p* value	
						Incubation						
		*cagA*	*babA2*	*dupA*	*iceA*	0 h	1 h	24 h	0 h–1 h	1 h–24 h	0 h–1 h	1 h–24 h
6171/T.II	s2m2					2.49 ± 0.03 (0.83–5.25)	2.44 ± 0.05 (0.67–5.7)	1.5 ± 0.05 (0.53–3.84)	− 2.01	− 38.52**	1.06; 0.2899	12.58; 0.0000
7361/T.II					+	2.43 ± 0.03 (0.83–5.24)	2.4 ± 0.03 (0.72–6.44)	1.42 ± 0.03 (0.5–4.48)	− 1.23	− 40.83**	1.23; 0.2179	10.63; 0.0000
7104/T.III		+			+	2.37 ± 0.04 (0.75–6.67)	2.18 ± 0.04 (0.54–4.76)	1.48 ± 0.03 (0.53–3.67)	− 8.02*	− 32.11**	2.52; 0.017	10.26; 0.0000
7357/T.II				+	+	2.31 ± 0.04 (0.67–5.07)	2.13 ± 0.02 (0.59–6.89)	1.57 ± 0.02 (0.51–7.18)	− 7.79**	− 26.29**	2.94; 0.0033	6.50; 0.0000
7433/T.II			+	+		2.25 ± 0.02 (0.69–5.03)	2.02 ± 0.03 (0.5–5.43)	1.52 ± 0.03 (0.5–3.41)	− 10.22**	− 24.75**	3.69; 0.0002	7.87; 0.0000
6885/T.III	s1m2					2.26 ± 0.04 (0.69–4.47)	1.86 ± 0.04 (0.55–3.86)	1.47 ± 0.03 (0.54–3.43)	− 17.7**	− 20.97**	6.77; 0.0000	7.38; 0.0000
7042/T.I		+				2.24 ± 0.04 (0.7–4.4)	1.7 ± 0.02 (0.5–11.5)	1.37 ± 0.02 (0.5–5.93)	− 24.11**	− 19.41	7.25; 0.0000	1.11; 0.2683
5530/T.I		+		+		2.2 ± 0.03 (0.69–4.42)	1.53 ± 0.04 (0.57–4.64)	1.31 ± 0.03 (0.52–4.42)	− 30.45**	− 14.38**	8.83; 0.0000	3.17; 0.0015
7317/T.I		+		+	+	2.18 ± 0.04 (0.67–4.25)	1.51 ± 0.04 (0.52–5.57)	1.2 ± 0.03 (0.51–4.15)	− 30.73**	− 20.53**	9.26; 0.0000	4.79; 0.0000
7297/T.I		+	+		+	2.17 ± 0.03 (0.69–4.98)	1.46 ± 0.02 (0.54–4.66)	1.31 ± 0.04 (0.52–3.71)	− 32.72**	− 10.27*	10.26; 0.0000	2.78; 0.0055
7101/T.I	s1m1	+			+	2.12 ± 0.02 (0.67–4.76)	1.3 ± 0.04 (0.5–3.34)	1.25 ± 0.02 (0.5–4.26)	− 38.68**	− 3.85	11.5; 0.0000	0.89; 0.3733
7143/T.I		+	+			2.15 ± 0.03 (0.68–4.36)	1.21 ± 0.02 (0.5–3.1)	1.18 ± 0.04 (0.5–3.46)	− 43.72**	− 2.48	12.57; 0.0000	0.32; 0.7481
6237/T.I		+		+	+	2.13 ± 0.03 (0.6–3.99)	1.19 ± 0.03 (0.53–3.9)	1.14 ± 0.03 (0.5–2.61)	− 44.13**	− 4.20	12.77; 0.0000	1.62; 0.1058
J99		+	+	+	+	2.1 ± 0.03 (0.7–3.98)	1.02 ± 0.02 (0.52–2.1)	0.9 ± 0.04 (0.5–1.85)	− 51.43**	− 11.76**	13.86; 0.0000	4.69; 0.0000

All *H. pylori* strains are indicated by their collection number and virulence type, where T.I indicates type I strains, T.II indicates type II strains and T.III indicates type III strains. *vacA* polymorphism column: s2m2 means non-toxic variants of this gene; s1m1 and s1m2 indicate variants of high and intermediate toxic variants of this gene, respectively. In columns *cagA*/*babA2*/*dupA*/*iceA*: + indicates strain containing the gene, free space indicates strain lacking the gene. †Reduction of cell length between 0 h (a), 1 h (b), and 24 h (c) incubation periods was measured using following formula: the interval of 0 to 1 h = (b ∕ a − 1) × 100% and the interval of 1 to 24 h = (c ∕ b − 1) × 100%. Asterisk and double asterisk indicates results that were significant (*p* < 0.05) and highly significant (*p* < 0.005) using the Wilcoxon *Z* test

The statistical analysis of the reduction in bacterial cell size with a specific virulence factor was found to be significant for *vacA* polymorphism (*p* = 0.0039) and the presence of *cagA* (*p* = 0.0113) and *babA2* (*p* = 0.0179). This significance was not observed for *dupA* (*p* = 0.8465) and *iceA* (*p* = 0.9485) genes.

The reduction rate of cell length during incubation from 0 to 1 h was positively correlated with the presence of virulence genes (Table 2). The reduction of bacterial cell size counted for 38.68–51.54% (*p* < 0.005), 17.7–32.72% (*p* < 0.005), and 2.01–10.22% (*p* < 0.05, except from 6171/T.II and 7361/T.II strains) for *vacA*s1m1, *vacA*s1m2, and *vacA*s2m2 strains, respectively. The opposite situation was observed during the incubation of bacteria from 1 to 24 h. The reduction rate in size was inversely correlated with the presence of virulence genes. In *vacA*s2m2 and *vacA*s1m2 strains, the ranges were 24.75–40.83% *(p* < 0.005) and 10.27–20.97% (*p* < 0.05, except for 7042/T.I strain), respectively. In the case of the strain J99 (*vacA*s1m1), a distinct reduction in cell size was observed (*p* < 0.005), but among other *vacA*s1m1 strains, the decrease in cell length ranged from 2.48 to 4.2% and was insignificant (*p* > 0.05).

### Analysis of cell shape variability

**Fig. 2:**
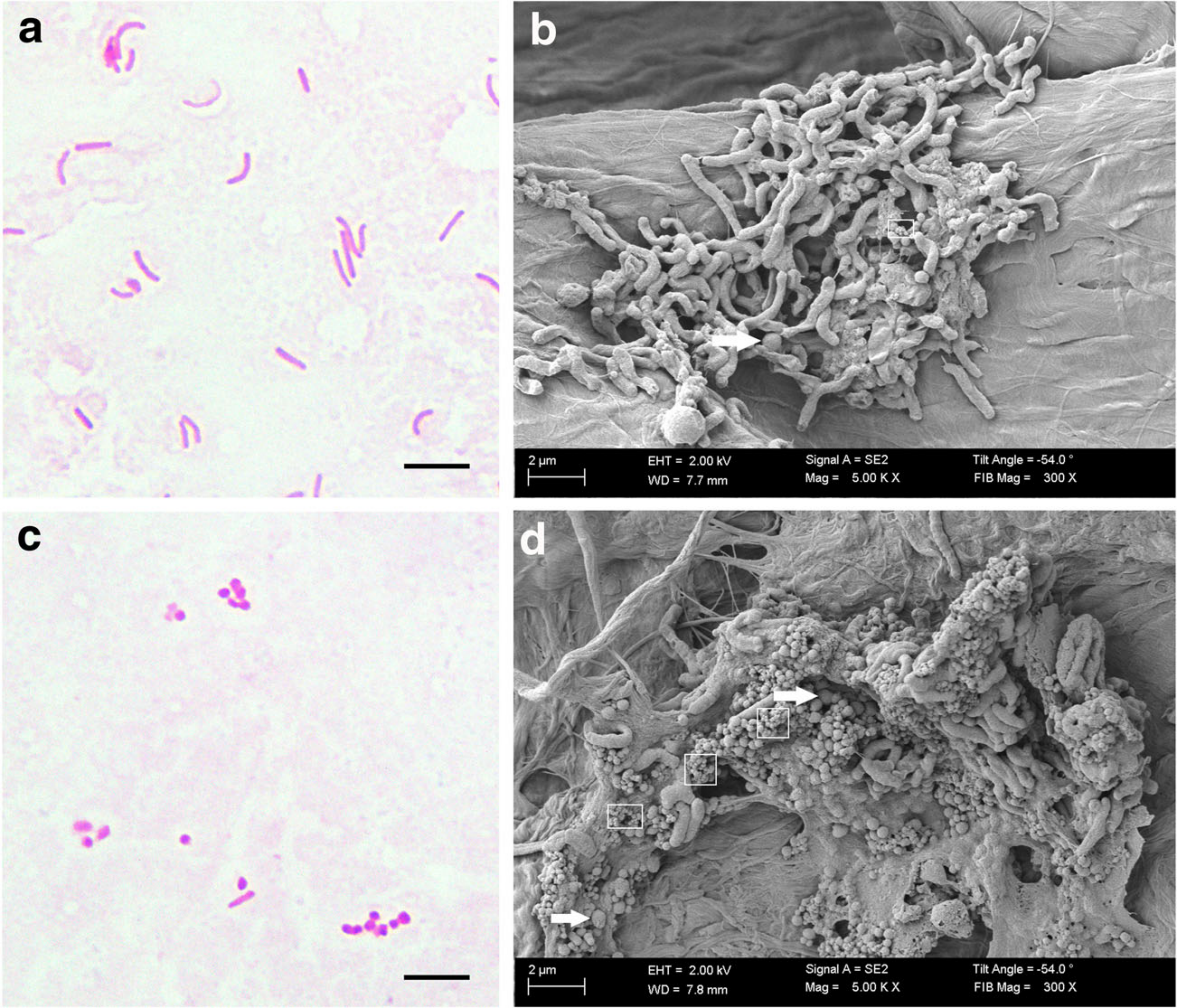
Differences in cell morphology of *H. pylori* strains observed under light and scanning electron microscope. Bacteria cultured for 1 h in serum-free BHI broths at suboptimal conditions (room temperature and atmosphere, without shaking). The *H. pylori* 6171/T.II strain seen mainly as rods (type II, low virulence) under light microscopy (**a**) and SEM (**b**), and *H. pylori* J99 seen mainly as coccoid forms (type I, high virulence) under light microscopy (**c**) and SEM (**d**). Coccoid forms of *H. pylori* are marked by arrows, while the small spherical structures within the squares indicate outer membrane vesicles. Scale bar in light microscopy, 5 μm

Table 3 shows the morphological alternations of all examined *H. pylori* strains after 1-h incubation under stressogenic culture conditions. Strain 6171/T.II with low virulence (*vacA*s2m2, *cagA*^−^, *dupA*^−^, *iceA*^−^, *babA2*^−^) at 1-h incubation was predominantly in the form of rods (61.3%), but also produced elongated/ filamentous cells (5.7%), short rods (29.3%), and coccoid forms (3.7%) (Fig. 2). A completely different phenotype was demonstrated for a highly pathogenic strain, namely *H. pylori* J99 (*vacA*s1m1, *cagA*^+^, *dupA*^+^, *iceA2*^+^, *babA2*^+^). Under the same conditions, it mainly formed coccoids (56.3%) and short rods (42.3%), while rod forms counted for only 1.3%, and elongated/ filamentous forms were not observed (Fig. 2).

**Table 3:** Morphological features of *H. pylori* type I strains in comparison to type II and III strains after 1-h incubation period in stressogenic culture conditions

Cell morphology	Distribution (%)													
	6171/T.II	7361/T.II	7357/T.II	7433/T.II	7104/T.III	6885/T.III	7042/T.I	5530/T.I	7317/T.I	7297/T.I	7101/T.I	7143/T.I	6237/T.I	J99
	*vacA*s2m2				*vacA*s2m2	*vacA*s1m2	*vacA*s1m2				*vacA*s1m1			
Coccoid forms	3.7	10.3	8.3	15.3	10.3	8.0	*48.3*	*31.3*	*34.0*	*29.7*	*43.7*	*50.7*	*39.3*	*56.3*
Short rods	29.3	32.0	33.0	38.7	41.7	54.7	26.7	45.7	43.3	52.0	42.7	36.0	55.0	42.3
Rods	61.3	47.7	56.3	38.7	46.0	37.3	17.0	21.7	21.0	17.3	13.7	13.4	5.7	1.3
Elongated rods	4.7	6.0	2.3	5.0	1.3	0.0	2.3	1.3	1.3	1.0	*0.0*	*0.0*	*0.0*	*0.0*
Filamentous forms	1.0	4.0	0.0	2.3	0.7	0.0	5.7	0.0	0.3	0.0	*0.0*	*0.0*	*0.0*	*0.0*

The profile of virulence factors and *vacA* polymorphisms that are associated with virulence types are referred to Table 2. Italicized values show the characteristic morphological features of tested *H. pylori* strains, allowing the classification into the relevant virulence types (II, III, I s1m2, I s1m1) during the screening microscopic observation in short period

**Fig. 3:**
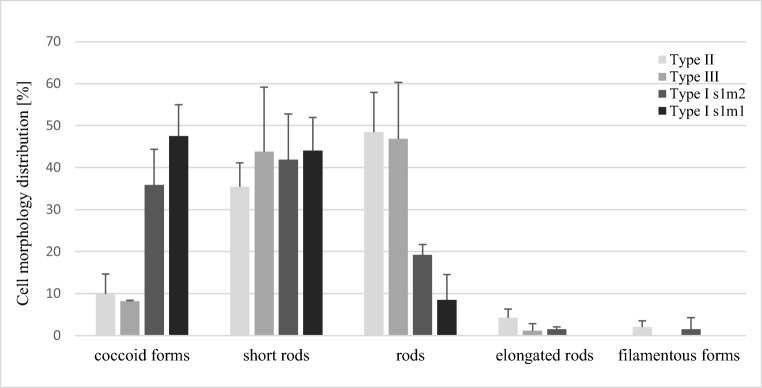
The impact of virulence type on the morphology distribution of *H. pylori* strains after 1-h incubation period in stressogenic culture conditions. Cell morphology measured from light microscopy images reports as percent of the total bacterial population examined. Data combined from two independent bacterial cultures incubated in serum-free BHI broths at suboptimal culture conditions for 1 h period (*n* = 300 cells/strain). Results are given as mean ± SDM (*n* = 6)

For analyzing the differences in the morphological properties, tested strains were classified into four groups, namely type II, type III, type I s1m2 (*cagA*^*+*^, *vacA*s1m2), and type I s1m1 (*cagA*^*+*^, *vacA*s1m1). After 1-h incubation, the presence of several times higher numbers of spherical forms in *H. pylori* type I strains (35.83% and 47.5% for type I s1m2 and I s1m1, respectively) than in type III (8.17%) (*p* < 0.000) and II (9.92%) (*p* < 0.000) strains was demonstrated (Fig. 3). The inverse relationship was observed for the number of rods, which were more common in type III (46.83%) and II (48.42%) strains than in type I s1m2 (19.25%) (*p* < 0.000) or I s1m1 (6.58%) (*p* < 0.000) strains (Fig. 3). Despite no statistically significant differences in the morphological properties of *H. pylori* type I s1m1 and I s1m2 strains (*p* = 0.141), the existence of a feature distinguishing both types was noted. In the case of *H. pylori* type I s1m1 strains, filamentous/elongated forms were never observed, while in *H. pylori* type I s1m2 strains, these forms were present and counted for 3% of all examined cells (Table 3).

## Discussion

*H. pylori* in the natural habitat is mainly present as spiral forms, whereas culture in artificial in vitro conditions or exposure to suboptimal environmental factors is accompanied by morphological transformation into spherical or filamentous forms (Azevedo et al. 2007; Cellini et al. 2008; Park et al. 2011; Shao et al. 2012; Faghri et al. 2014). This pleomorphic nature is not specific to *H. pylori* only, since the ability to undergo morphological transformation has been observed for many Gram-negative rods, i.e. *Campylobacter jejuni*, *Enterobacter aerogenes*, *Escherichia coli*, *Klebsiella pneumoniae*, *Legionella pneumophila*, *Proteus mirabilis*, *Salmonella enterica*, and *Vibrio cholerae* (Krogfelt et al. 1993; Justice et al. 2006, 2014; Chaiyanan et al. 2007; Krebs and Taylor 2011; Horvath Jr et al. 2012; Ramamurthy et al. 2014; Ghaffar et al. 2015).

The current study has found that adaptational changes in cell morphology were closely related to the virulence profile of *H. pylori* strains. Rossetti et al. (2013) postulated that increased cell size (including the intensification of the filamentation process) is potentially a feature of commensal microbes, while reduction of elongation intensity can be traced as a pathogenic hallmark. This hypothesis is in agreement with the results obtained in our study. We noted that lower virulence strains had longer cells than highly virulent strains. Moreover, the reduction in cell size of the tested *H. pylori* strains during short-term exposure to suboptimal conditions was positively correlated with the presence of certain virulence genes, e.g., *vacA*, *cagA*, and *babA*.

It seems that strains with higher adaptability (and higher virulence) are capable of achieving reduced cell size in a shorter time during exposure to stressogenic conditions. The results in this study, showing that *H. pylori* strains with a higher virulence potential are capable of morphological adaptations in a short period to adverse environmental conditions, are consistent with reports by Vitoriano et al. (2011). The authors undertook co-culture experiments of peptic ulcer disease (PUD) and nonulcer dyspepsia (NUD) *H. pylori* strains with eukaryotic cell lines. It was demonstrated that after 1 day of co-culture, NUD strains possessed their original spiral shape, whereas PUD strains obtained spherical form as early as after 12-h co-incubation time. It has been suggested that this phenomenon is caused by the more severe destruction of eukaryotic cells and necrosis-dependent changes in environmental conditions and thus faster transformation of *H. pylori* PUD strains into coccoid forms. There are also scientific reports suggesting that minimization of bacterial cell size is a protective feature that contributes to the reduction of the cellular surface exposed to the immune system clearance (complement deposition or opsonophagocytosis) (Dalia and Weiser 2011; Veyrier et al. 2015). Their suggestions are within the scope of our observations showing that under suboptimal conditions (including nutrient depletion or exposure to the oxygenic and thermic stress), the process of reducing cell size and creating short rods or coccoid forms may be a potentially adaptive feature.

The direct molecular mechanism underlying the relationship between the presence of virulence genes and *H. pylori* morphology is not known. The presence of *H. pylori* virulence genes, however, does not have to exert a direct influence on the bacterial phenotype. Modulation of the morphology or physiology of microbial cells could also have an indirect impact. For example, there is a strong relationship between the presence of *dupA* and *H. pylori* growth at low pH (Lu et al. 2005; Talebi Bezmin Abadi et al. 2012). Bacterial adaptation in different pH conditions may contribute to the activation or inhibition of different molecular mechanisms. There are many proteins that become active or inactive depending on various pH environments. In a similar fashion, the phenotype of *H. pylori* may be influenced by other environmental factors. The infection caused by *H. pylori* strains, especially those producing many virulence factors, contributes to the formation of the inflammatory altered gastric environment, which is characterized by occurrence of numerous immune cells (neutrophils, macrophages, and T cells), proinflammatory cytokines (IL-1β, IL-6, IL-8, and TNF-α), chemokines, and matrix metalloproteinases (Ma et al. 2016). Survival in the inflamed environment requires the presence of multiple genes that protect against the immune clearance, which are most likely co-expressed with the expression of virulence genes, such as *cagA*, *vacA*, or *babA*. Some of these genes, protecting against immune attacks and the presence of unfavorable environmental conditions, could encode information about factors responsible for rapid morphological changes in *H. pylori*.

### Limitations

Our study has some limitations. Firstly, the analysis was conducted with selected virulence genes (*cagA*, *vacA*, *babA2*, *dupA*, *iceA*). The selection of these virulence determinants was dictated by literature data indicating the significance of the abovementioned genes in the development of gastrointestinal disorders (Zambon et al. 2003; Torres et al. 2009; Shiota et al. 2010, 2012). However, it cannot be ruled out that the presence of other genes, not investigated in this study, also influences *H. pylori* morphology. On the other hand, this method seems to be surprisingly sensitive because it detected differences in cell morphology in relatively low amounts of tested virulence determinants. Secondly, the analysis was performed with s1/2 and m1/2 *vacA* alleles only. The relevance of the *vacA* intermediate (i) region in the cytotoxic activity of VacA has been demonstrated (Rhead et al. 2007). In the current study, *H. pylori* strains were categorized into three groups, namely types I, II, and III. This classification does not include the i-region, but only the s- and m-regions (Xiang et al. 1995). Hence, the impact of this region has not been investigated. The link between the morphology of *H. pylori* cells and the presence of virulence genes needs to be explored in different populations.

## Conclusion

Based on microscopic observations, we demonstrated that the morphology of *H. pylori* strains might be influenced by the virulence profile. Less virulent *H. pylori* strains had a lower potential for the formation of coccoid forms than highly pathogenic strains. Furthermore, strains with lower pathogenicity were characterized by the presence of filamentous/ elongated forms, not observed in highly virulent strains.
